# Trends in Off-Label Indications of Non-Vitamin K Antagonist Oral Anticoagulants in Acute Coronary Syndrome

**DOI:** 10.31083/j.rcm2406180

**Published:** 2023-06-25

**Authors:** Rasha Kaddoura, Bassant Orabi, Mohamed A Yassin, Amr S Omar

**Affiliations:** ^1^Pharmacy Department, Heart Hospital, Hamad Medical Corporation, 3050 Doha, Qatar; ^2^Department of Hematology, National Center for Cancer Care and Research, Hamad Medical Corporation, 3050 Doha, Qatar; ^3^Department of Cardiothoracic Surgery/Intensive Care Unit, Heart Hospital, Hamad Medical Corporation, 3050 Doha, Qatar

**Keywords:** ACS, CCS, chronic coronary syndrome, DOACs, LV thrombus, myocardial infarction, NOACs, stent thrombosis

## Abstract

Acute coronary syndrome (ACS) is a leading cause of mortality worldwide. Despite optimal antiplatelet therapy recommendation after ischemic events, recurrent thrombotic complications rate remains high. The recurrent events maybe in part due to increased thrombin levels during ACS which may underscore the need for an additional anticoagulation therapy. Given the advantages of non-vitamin K antagonist oral anticoagulants (NOACs) over warfarin, they have the potential to prevent thrombus formation, in the presence or absence of atrial fibrillation, but at the cost of increased risk of bleeding. NOACs have also shown a promising efficacy in managing left ventricular thrombus and a potential benefit in avoiding stent thrombosis after percutaneous coronary revascularization. Taken as a whole, NOACs are increasingly used for off-licence indications, and continue to evolve as essential therapy in preventing and treating thrombotic events. Herein, this review discusses NOACs off-label indications in the setting of ischemic coronary disease.

## 1. Introduction

Acute coronary syndrome (ACS) is a medical emergency that occurs because of coronary artery occlusion leading to myocardial hypoperfusion [1]. ACS is associated with morbidity and mortality, particularly during hospitalization and 30 days after the event. However, the risk of recurring cardiovascular events persists beyond that period. The history of anticoagulation agents to treating ACS started since 1930s in animal studies with intravenous heparin proven to reduce formation of thrombus. Further clinical studies followed in 1940s with the use of oral anticoagulants [2]. The introduction of non-vitamin K antagonist oral anticoagulants (NOACs) has revolutionized the landscape of anticoagulation therapy [3], and NOACs have become the cornerstone in the management of thrombosis in various cardiovascular contexts [4]. NOACs are either direct thrombin inhibitors, namely dabigatran, or factor Xa inhibitors, including apixaban, betrixaban, edoxaban, and rivaroxaban, that are characterised by predictable pharmacokinetic properties, quick action at onset and offset, fixed dosing-regimen, less frequent monitoring or follow-up needs, acceptable safety profile, few drug-food and drug-drug interactions, and comparable safety and efficacy with warfarin in the approved indications, i.e., atrial fibrillation (AF) and venous thromboembolism [4, 5]. In addition to the potential cost-saving benefit on the long run [5]. Consequently, NOACs were labelled for many indications by regulatory bodies and recommended by international guidelines [3], hence there was a growing interest in NOACs, and their use has been increasing in several off-label indications. This review discusses the off-label indications of NOACs in ischemic coronary disease such as in peri-percutaneous coronary procedures, post cardiac and non-cardiac surgeries, and left ventricular (LV) thrombus following ischemic events.

## 2. Simplistic Mechanism of Ischemic Coronary Thrombosis

**Fig. 1.**
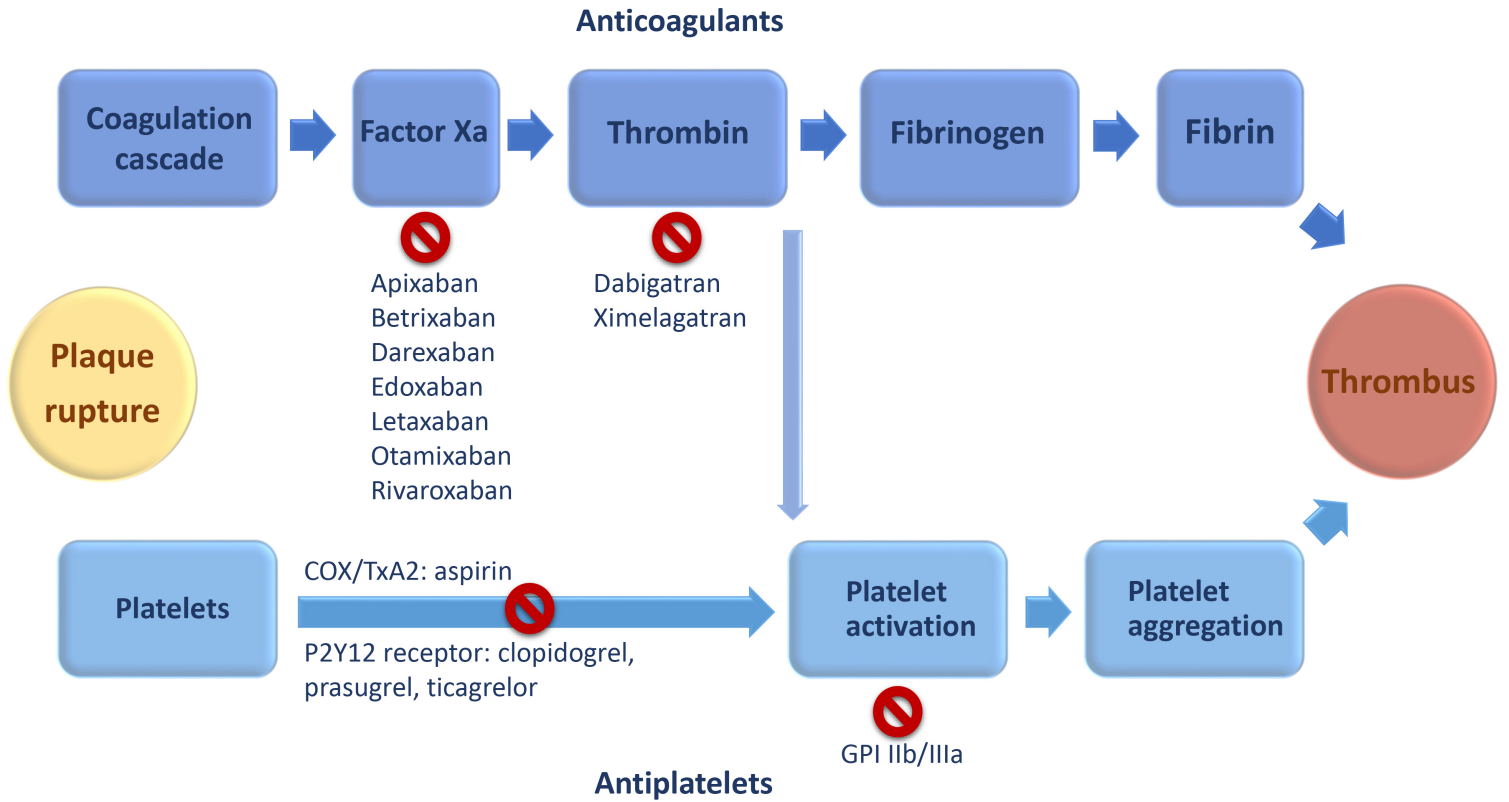
**Simplistic scheme of non-vitamin K antagonist oral anticoagulation and antiplatelet agents targets**. Abbreviations: COX, cyclooxygenase; GPI, glycoprotein inhibitors; TxA2, thromboxane A2.

Ischemic heart disease is a top cause of death, with 30% of deaths are caused by coronary artery disease (CAD) worldwide. Coronary thrombosis in patients with CAD leads to ACS and death [6, 7]. Patients presenting with ACS require immediate antithrombotic therapy [1]. The acute ischemic event occurs due to plaque rupture leading to coronary artery occlusion, either partially or totally. Then the vascular damage exposes von Willebrand factor (i.e., tissue factor) and collagen [7] to platelets which adhere to both collagen and von Willebrand factor in the ruptured plaque [2], with eventual platelet activation and aggregation [8]. The sub-endothelium-released tissue factor provokes the coagulation cascade and, subsequently, thrombin release which is implicated in thrombus formation and further platelet activation [2, 6, 7]. Anticoagulation agents act at several stages of the coagulation cascade to prevent thrombus formation and eventually new or recurrent thrombotic event [3]. Thus, the need for antithrombotic therapy (i.e., anti-platelet and anticoagulation agents) in ACS. NOACs have specific targets (factor Xa and thrombin) in the coagulation cascade, hence reducing the formation and progression of thrombus (Fig. 1) [7, 9].

**Fig. 2.**
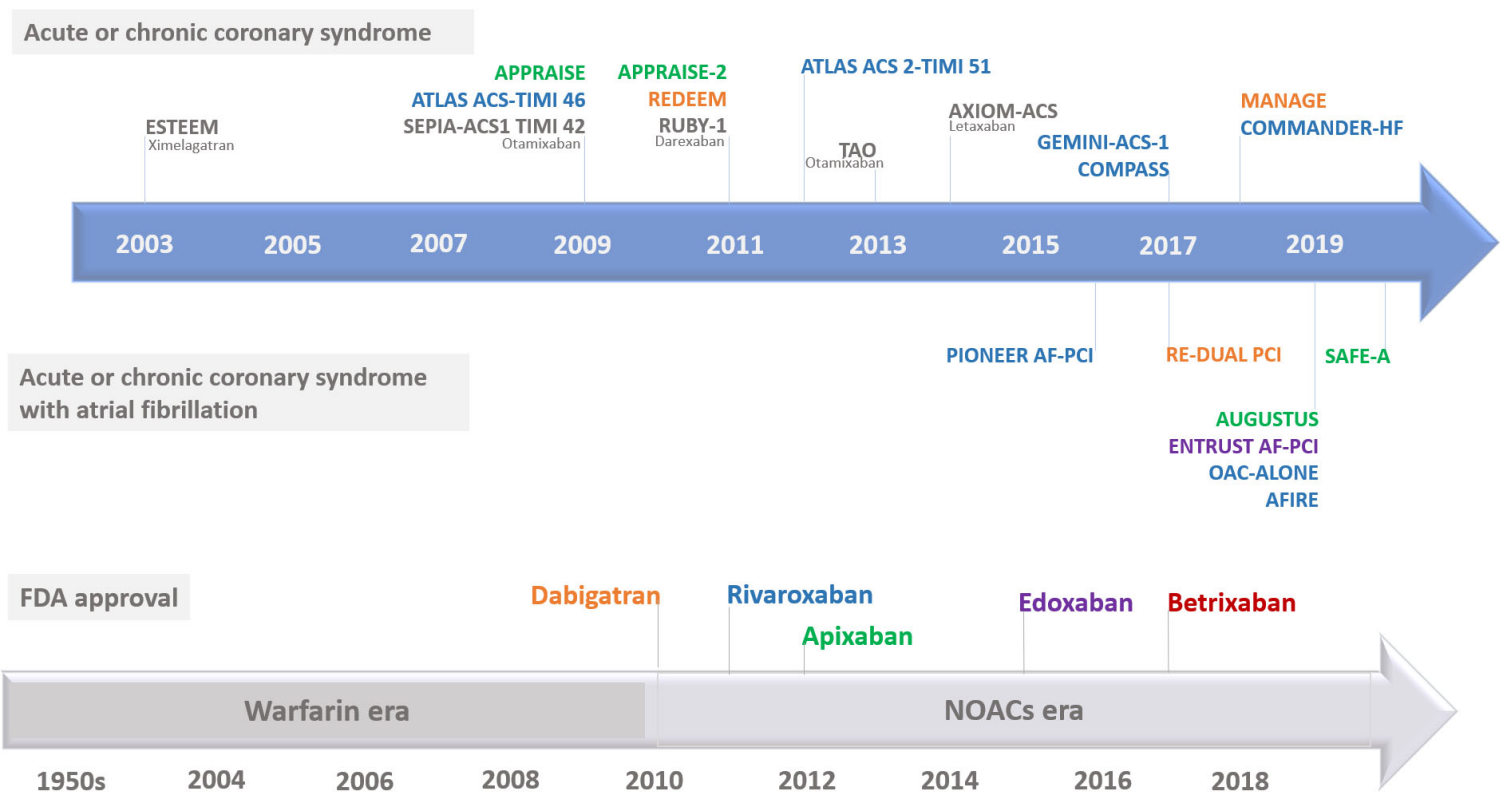
**Timeline of non-vitamin K antagonist oral anticoagulants approval and key studies**. FDA, Food and Drug Administration; NOACs, non-vitamin K antagonist oral anticoagulants.

Dual antiplatelet therapy (DAPT) by combing aspirin and a ${\mathrm{P2Y}}_{12}$ receptor inhibitor, is recommended for secondary prevention of recurrent events after ACS, and despite the benefit, the rate of recurrent cardiovascular events within 12 months may range from 9% to 12% [2, 7, 10]. When the duration of DAPT is prolonged beyond 12 months, it provides only minimal thromboembolic prevention without reduction in mortality risk [6]. The thrombotic risk may be attributed to the elevated levels of thrombin and factor Xa by-products that linger for weeks or months after the acute coronary event, resulting in a prolonged hypercoagulable state [7]. Thus, it was plausible to consider adding an oral anticoagulation agent to DAPT (i.e., dual-pathway approach) for secondary prevention to reduce the level of thrombin and improve clinical outcomes [1, 2]. Very early studies investigated combining vitamin K antagonists (VKAs) with aspirin in patients with acute or chronic coronary syndromes and demonstrated reduction in thrombotic complications, but at the cost of elevated bleeding risk [11, 12, 13]. NOACs may offer a supplemental role in managing ACS such as in the secondary prevention of cardioembolic events [6]. Table 1 summarizes the key characteristics of the approved NOACs [8, 14, 15], and Fig. 2 presents the timeline of NOACs approval and key studies that are discussed below.

**Table 1.** **General characteristics of the approved non-vitamin K antagonist oral anticoagulants**.

Group	Direct thrombin inhibitor	Factor Xa inhibitor		
Agent	Dabigatran	Apixaban	Edoxaban	Rivaroxaban
Approved indications	∙ AF (RE-LY)	∙ AF (ARISTOTLE, AVERROS)	∙ AF (ENGAGE AF-TIMI 48)	∙ AF (ROCKET AF)
	∙ VTE treatment (RE-COVER-I & -II)	∙ VTE treatment (AMPLIFY)	∙ VTE treatment (Hokusai-VTE)	∙ VTE treatment (EINSTEIN-DVT & -PE)
	∙ VTE secondary prevention (RE-SONATE)	∙ VTE secondary prevention (AMPLIFY-EXT)		∙ VTE secondary prevention (EINSTEIN-EXT)
	∙ Post-op VTE prophylaxis (RE-MODEL, RE-MOBILIZE, RE-NOVATE-I & -II)	∙ Post-op VTE prophylaxis (ADVANCE-1, -2 & -3)		∙ Post-op VTE prophylaxis (RECORD-1, -2, -3, & -4)
Dosing regimen	Twice daily	Twice daily	Twice daily	Once or twice daily
Bioavailability	3–7%	∼50%	62%	80%
Renal clearance	80%	27%	27–50%	33%
Prodrug	Yes	No	No	No
Half-life (hours)	12–18	8–14	9–11	7–11
Interaction	Substrate of P-gp efflux pump	Substrate of P-gp efflux pump	Substrate of P-gp efflux pump	Substrate of P-gp efflux pump
	Contraindicated with potent inhibitors of P-gp	Simultaneous strong CYP3A4 and P-gp inhibitors should be avoided	Avoid use with P-gp inducers/inhibitors	Simultaneous strong CYP3A4 and P-gp inhibitors should be avoided
	May interact with potent P-gp inducers			
Antidote	Idarucizumab	Andexanet alfa	Andexanet alfa	Andexanet alfa

Abbreviations: AF, atrial fibrillation; CYP, cytochrome 450 enzyme; DVT, deep vein thrombosis; PE, pulmonary embolism; P-gp, P-glycoprotein; post-op, post operative; VTE, venous thromboembolism; RE-LY, Randomized Evaluation of Long Term Anticoagulant Therapy.

## 3. Acute Coronary Syndrome 

The addition of NOACs to DAPT post ACS has been explored in various studies. The ESTEEM (efficacy and safety of the oral direct thrombin inhibitor ximelagatran in patients with recent myocardial damage) Phase II randomized study evaluated the first antithrombin NOAC to emerge, ximelagatran, in patients with ACS. Patients who received aspirin alone were randomized to receive ximelagatran or a placebo in four different doses. Ximelagatran significantly reduced composite of death, myocardial infarction and severe recurrent ischemia (i.e., primary endpoint) without significant bleeding episodes encountered between ximelagatran and placebo arms [16]. The ESTEEM sub-study showed that ximelagatran provided long-term thrombin generation reduction [17]. The drug was withdrawn from usage due to significant side effects on the liver [16]. In another sub-study, ximelagatran was found to be associated with reduction in D-dimer which is linked to cardiovascular complications [18]. Dabigatran was tested in the REDEEM (Randomized Dabigatran Etexilate Dose Finding Study in Patients with Acute Coronary Syndromes Post Index Event with Additional Risk factors for Cardiovascular Complications Also Receiving Aspirin and Clopidogrel) Phase II trial as an add-on to DAPT after ACS events. The bleeding episodes were significantly higher when compared to placebo, and the secondary outcome measures (all-cause death) were significantly lower with dabigatran [19]. The APPRAISE-1 (Apixaban for Prevention of Acute Ischemic and Safety Events) is a dose-finding study that randomized apixaban into four groups. The two groups of higher doses (20 mg once and 10 mg twice daily) were terminated early due of excessive bleeding. The study concluded a dose-associated increased bleeding with only a trend to decreased ischemic episodes. Apixaban as an add-on to aspirin plus clopidogrel caused more bleeding and less benefit in term of reducing ischemic events in comparison with aspirin alone [20]. Further examination of apixaban followed in Phase III APPRAISE-2 trial which was terminated early because of excessive bleeding events without significant benefit in terms of recurrent ischemic events [21]. The conclusion of the APPRAISE-2 trial did not change when the findings were analysed according to the background dual or single antiplatelet therapy [22]. Further analysis of the bleeding events in APPRAISE-2 trial demonstrated that apixaban increased both short- and long-term bleeding complications. The most frequent source of bleeding was the gastrointestinal tract [23].

The ATLAS ACS-TIMI (Anti-Xa Therapy to Lower Cardiovascular Events in Addition to Standard Therapy in Subjects with Acute Coronary Syndrome-Thrombolysis In Myocardial Infarction) Phase II (ATLAS ACS-TIMI 46) and III (ATLAS ACS 2-TIMI 51) trials showed that rivaroxaban reduced major ischemic episodes with a dose-dependent elevated bleeding risk [24, 25]. Several analyses of the ATLAS ACS 2-TIMI 51 trial have been performed. Rivaroxaban showed benefit in reducing cardiovascular episodes which appeared early and maintained during the treatment without significant rise in fatal bleeding [26]. The majority of myocardial infarction events, i.e., endpoints in ACS patients after stabilization, were spontaneous, rivaroxaban significantly reduced them especially those associated with ST-segment elevation and substantial release of cardiac biomarkers [27]. The use of 2.5-mg dose had more favourable safety and efficacy outcomes than 5-mg dosing regimen [28]. A meta-analysis of four studies by Yuan and colleagues [29] (n = 40,148) found that combining rivaroxaban with antiplatelet therapy in patients presenting with ACS, was an effective strategy but with a doubtful safety benefit. In the United States, unlike in Europe, the Food and Drug Administration has not labelled add-on rivaroxaban after ACS for secondary prevention despite the reported benefit because of the large burden of missing data in ATLAS ACS 2-TIMI 51 trial [30]. Moreover, the increased risk of bleeding rendered this strategy to be scarcely used. Komócsi *et al*. [31] pooled the results of seven randomized trials (n = 31,286) that used NOACs on top of antiplatelet therapy in ACS patients and found a significant increase in major bleeding by three folds (odds ratio (OR) 3.03; 95% CI: 2.20–4.16) without overall mortality or net clinical (i.e., composite of ischemic and major bleeding events) benefits. When rivaroxaban was combined with a ${\mathrm{P2Y}}_{12}$ receptor inhibitor instead of aspirin, the GEMINI-ACS-1 (Randomized, Double-Blind, Double-Dummy, Active-Controlled, Parallel-group, Multicenter Study to Compare the Safety of Rivaroxaban Versus Acetylsalicylic Acid in Addition to Either Clopidogrel or Ticagrelor Therapy in Subjects With Acute Coronary Syndrome) trial concluded that low-dose rivaroxaban (i.e., 2.5 mg twice daily) had comparable bleeding or safety profile to DAPT [32].

Edoxaban was not studied in combination with antiplatelet therapy in ACS patients. Darexaban combined with DAPT in the RUBY-1 (randomized, double blind, placebo-controlled trial of safety and tolerability novel oral factor Xa inhibitor darexaban (YM150) following acute coronary syndrome) Phase II randomized trial significantly increased bleeding risk without an observed benefit in lowering cardiovascular events. Thus, further investigation with darexaban was put on hold by the manufacturer [33]. With regards the impact of antiplatelet therapy, Khan *et al*. [34] in their meta-analysis found that combining NOACs with single antiplatelet agent did not decrease ischemic episodes or increased bleeding complications. Whereas, adding NOACs to DAPT significantly increased bleeding (hazard ratio (HR) 2.24; 95% CI: 1.75–2.87) and modestly reduced major adverse cardiovascular events (HR 0.86; 95% CI: 0.78–0.93). On the other hand, Oldgren and colleagues [35] pooled efficacy and safety outcomes from the trials discussed above (ESTEEM, REDEEM, APPRAISE-1, APPRAISE-2, ATLAS ACS-TIMI 46, ATLAS ACS 2-TIMI 51, GEMINI-ACS-1, RUBY-1) and demonstrated that combining NOACs with dual or single antiplatelet therapy significantly reduced major adverse cardiovascular events [(HR 0.70; 95% CI: 0.59–0.84) or (HR 0.87; 95% CI: 0.80–0.95), respectively], but the combination with either antiplatelet therapy regimen caused more clinically significant bleeding events [(HR 1.79; 95% CI: 1.54–2.09) and (HR 2.34; 95% CI: 2.06–2.66), respectively]. Heterogeneity was low between the trials, and the results did not differ when the analysis was restricted to Phase II trials [35]. Otamixaban was tested in the SEPIA-ACS1 TIMI 42 (Study Program to Evaluate the Prevention of Ischemia with direct Anti-Xa inhibition in Acute Coronary Syndromes 1—Thrombolysis in Myocardial Infarction 42) phase II study against heparin plus eptifibatide in non-ST-segment elevation myocardial infarction. Parenteral otamixaban use showed a trend towards lowering ischemic episodes without a difference in safety outcomes between the two arms [36]. Subsequently, TAO (Treatment of Acute Coronary Syndromes with Otamixaban) Phase III trial did not confirm any benefit of otamixaban in decreasing ischemic episodes rate but found an increase in bleeding events [37]. Finally, the factor Xa inhibitor letaxaban, in AXIOM ACS (Safety and efficacy of TAK-442 in subjects with acute coronary syndromes) Phase II dose-ranging randomized trial, was tested for tolerability and safety. As compared with placebo, letaxaban in varying doses did not increase major bleeding rate (i.e., primary endpoint) or improve efficacy endpoint [38]. There was no further testing of this agent in ACS. The summary of the key studies is shown in Table 2 (Ref. [19, 20, 21, 24, 25, 32]).

**Table 2.** **Non-vitamin K antagonist oral anticoagulants for acute and chronic coronary syndrome**.

Study	Population	Intervention and Comparator(s)	Efficacy	Safety
NOACs vs comparator				
Acute coronary syndrome				
Oldgren *et al*. 2011 (REDEEM) [19]	∙ Patients with STEMI or NSTEMI	∙ Dabigatran 50, 75, 110, or 150 mg BID plus DAPT	Death	Bleeding – HR (95% CI)
RCT, Phase II		∙ Placebo plus DAPT	∙ 50 mg: 2.2%; 75 mg: 2.7%	∙ 50 mg: 3.5%; 1.77 (0.70, 4.50)
N = 1861			∙ 110 mg: 1.7%; 150 mg: 2.0%	∙ 75 mg: 4.3%; 2.17 (0.88, 5.31)
			∙ Placebo: 3.8%	∙ 110 mg: 7.9%; 3.92 (1.72, 8.95)
			Thrombotic events:	∙ 150 mg: 7.8%; 4.27 (1.86, 9.81)
			∙ 50 mg: 4.6%; 75 mg: 4.9%	∙ Placebo: 2.2%
			∙ 110 mg: 3.0%; 150 mg: 3.5%	
			∙ Placebo: 3.8%	
			D-dimer reduced in dabigatran dose groups (*p* = 0.001)	
Alexander *et al*. 2009 (APPRAISE-1) [20]	∙ Patients with STEMI or NSTEMI	∙ Apixaban 2.5 mg BID, 10 mg OD, 10 mg BID, or 20 mg OD plus APT	Ischemic events	Total bleeding – HR (95% CI)
RCT, Phase II		∙ Placebo plus APT	∙ 2.5 mg BID: 7.6%; 0.73 (0.44, 1.19)	∙ 2.5 mg BID 1.6%; 1.78 (0.91, 3.48)
N = 1715			∙ 10 mg OD: 6.0%; 0.61 (0.35, 1.04)	∙ 10 mg OD: 1.9% 2.45 (1.31, 4.61)
				∙ 10 mg or 20 mg OD: excess total bleeding led to both arms discontinuation
				More bleeding when on DAPT
Alexander *et al*. 2011 (APPRAISE-2) [21]	∙ Patients with STEMI or NSTEMI	∙ Apixaban 5 mg BID plus APT	∙ CV death, MI, or ischemic stroke: 7.5% vs 7.9%; HR 0.95 (95% CI: 0.80, 1.11)	∙ Premature trial termination due to major bleeding
RCT		∙ Placebo plus APT	∙ Recurrent ischemic events: no reduction in rate	∙ Major bleeding: 1.3% vs 0.5%; HR 2.59 (95% CI: 1.50, 4.46)
N = 7392				
Mega *et al*. 2009 (ATLAS ACS-TIMI 46) [24]	∙ Patients with ACS	∙ Rivaroxaban 5, 10, 15, or 20 mg per day (divided once or twice) plus APT	∙ Death, MI, stroke, or severe recurrent ischemia: 3.9% vs 5.5%; HR 0.69 (95% CI: 0.50, 0.96)	Significant bleeding – HR (95% CI)
RCT, Phase II				∙ 5 mg: 6.1%; 2.21 (1.25, 3.91)
N = 3491				∙ 10 mg: 10.9%; 3.35 (2.31, 4.87)
				∙ 15 mg: 12.7%; 3.60 (2.32, 5.58)
				∙ 20 mg: 15.3%; 5.06 (3.45, 7.42)
Mega *et al*. 2012 (ATLAS ACS 2-TIMI 51) [25]	∙ Patients with ACS	∙ Rivaroxaban 2.5, 5 mg BID	CV Death, MI, or stroke: 8.9% vs 10.7%; HR 0.84 (95% CI: 0.74, 0.96)	∙ Rivaroxaban increased the rates of major bleeding not related to CABG (2.2% vs 0.6%; HR 4.52 (95% CI: 2.27, 9.01)
RCT		∙ Placebo	∙ 2.5 mg: 9.1% vs 10.7%, *p *= 0.02	
N = 15,526			∙ 5 mg: 8.8% vs 10.7%, *p* = 0.03	
			2.5 mg only:	
			∙ CV death: 2.7% vs 4.1%, *p* = 0.002	
			∙ Death: 2.9% vs 4.5%, *p* = 0.002	
Ohman *et al*. 2017 (GEMINI-ACS-I) [32]	∙ Patients with STEMI or NSTEMI	∙ Rivaroxaban 2.5 mg BID plus ${\mathrm{P2Y}}_{12}$	∙ CV death, MI, stroke, or definite stent thrombosis: 5.0% vs 5.0%; HR 1.06 (95% CI: 0.77, 1.46)	∙ Clinically significant non-CABG bleeding: 5.0% vs 5.0%; HR 1.09 (95% CI: 0.80, 1.50)
RCT		∙ Aspirin plus ${\mathrm{P2Y}}_{12}$		
N = 3037				
Chronic coronary syndrome				
Eikelboom *et al*. 2017 (COMPASS) [39]	∙ Stable atherosclerotic vascular disease	∙ Rivaroxaban 2.5 mg BID plus aspirin	Rivaroxaban-plus-aspirin vs aspirin	Rivaroxaban-plus-aspirin vs aspirin
RCT		∙ Rivaroxaban 5 mg BID	∙ CV death, stroke, or MI: 4.1% vs 5.4%; HR 0.76 (95% CI: 0.66, 0.86)	∙ Major bleeding: 3.1% vs 1.9%; HR 1.70 (95% CI: 1.40, 2.05)
N = 27,395		∙ Aspirin	∙ Death: 3.4% vs 4.1%; HR 0.82 (95% CI: 0.71, 0.96)	∙ Fatal bleeding: no difference
			Rivaroxaban vs aspirin	Rivaroxaban vs aspirin
			∙ CV death, stroke, or MI: 4.9% vs 5.4%; HR 0.90 (95% CI: 0.79, 1.03)	∙ Major bleeding: 2.8% vs 1.9%; HR 1.51 (95% CI: 1.25, 1.84)
			∙ Death: 4.0% vs 4.1%; HR 0.97 (95% CI: 0.84, 1.12)	∙ Fatal bleeding: no difference
Zannad *et al*. 2018 (COMMANDER-HF) [43]	Chronic HF, LVEF ≤40%, CAD, NSR, elevated concentrations of natriuretic peptides	∙ Rivaroxaban 2.5 mg BID	∙ Death from any cause, MI, or stroke: 25% vs 26.2% HR 0.94; (95% CI: 0.84, 1.05)	∙ Fatal bleeding or bleeding into a critical space with a potential for causing permanent disability: 0.7% vs 0.9%; HR 0.80 (95% CI: 0.43, 1.49)
RCT		∙ Placebo		∙ Rivaroxaban group: more bleeding requiring hospitalization and are at higher risk of major bleeding
N = 5022				

Abbreviations: ACS, acute coronary syndrome; APT, antiplatelet therapy; BID, twice daily; CAD, coronary artery disease; CABG, coronary artery bypass grafting; CI, confidence interval; CV, cardiovascular; DAPT, dual antiplatelet therapy; HF, heart failure; HR, hazard ratio; LVEF, left ventricular ejection fraction; MI, myocardial infarction; NOACs, non-vitamin K antagonist oral anticoagulants; NSR, normal sinus rhythm; NSTEMI, non-ST segment elevation myocardial infarction; OD, once daily; RCT, randomized controlled trial; STEMI, ST-segment elevation myocardial elevation.

## 4. Chronic Coronary Syndrome 

NOACs have also been investigated as monotherapy or combined with antiplatelet therapy in stable ischemic or atherosclerotic diseases [8]. The large COMPASS (Cardiovascular OutcoMes for People Using Anticoagulation StrategieS) trial demonstrated that low-dose rivaroxaban (i.e., 2.5 mg twice daily) as add-on to aspirin significantly reduced composite cardiovascular events and mortality by 24% and 18%, respectively, in comparison with aspirin monotherapy but at the expense of significant rise in major bleeding events by 70%. When compared with aspirin alone, 5-mg twice daily rivaroxaban increased bleeding events without a difference in cardiovascular benefit [39]. Gastrointestinal tract (1–2%) was the most frequent source of major bleeding in the study participants [40]. Patients from international registries who were described to be eligible for enrolment in COMPASS trial experienced more cardiovascular adverse events than those participated in the trial [41, 42]. Given that the presence of heart failure may activate thrombin-associated pathways, it was hypothesized that rivaroxaban can decrease thrombin generation in patients who have underlying CAD and presenting with decompensated heart failure. In the COMMANDER-HF (A Study to Assess the Effectiveness and Safety of Rivaroxaban in Reducing the Risk of Death, Myocardial Infarction, or Stroke in Participants With Heart Failure and Coronary Artery Disease Following an Episode of Decompensated Heart Failure) trial, rivaroxaban (2.5 mg twice daily) did not significantly decrease cardiovascular complications in CAD patients presenting with decompensated heart failure [43]. A post-hoc analysis of COMMANDER-HF trial concluded that rivaroxaban decreased the rate of thromboembolic events (HR 0.83; 95% CI: 0.72–0.96) [44], and another analysis found that rivaroxaban reduced transient ischemic attack or stroke rates versus placebo (adjusted HR 0.68; 95% CI: 0.49–0.94) with similar bleeding rates [45]. The key two studies in chronic coronary syndrome are summarised in Table 2.

## 5. Acute Coronary Syndrome and Atrial Fibrillation

In acute or chronic coronary syndromes, AF is a common finding. Patients with AF could have five-fold increase in stroke which renders stroke prevention therapies such as anticoagulation, the cornerstone of therapy [46]. Among individuals with CAD, the reported prevalence of AF is 12.5% [8], and in ACS, the incidence of AF ranges from 2% to 23% [46]. Five to 10% of patients presenting with ACS have AF and using oral anticoagulation therapy [47]. Patients with AF and ACS have less favourable clinical outcomes [46, 48]. Patients with concurrent myocardial infarction and AF usually have higher stroke rate (3.1%) than those without AF (1.3%) [49]. As ACS requires DAPT, the presence of AF makes it a challenging scenario where healthcare providers must balance risks and benefits of the indicated triple antithrombotic therapy (TAT) with regards to prevention of ischemic episodes, stroke, stent thrombosis, systemic embolism, and bleeding [48].

Several randomized controlled trials evaluated NOACs in ACS patients undergoing percutaneous coronary intervention (PCI). These trials prespecified bleeding as the primary safety outcome and were not powered to ascertain ischemic benefits. The PIONEER AF-PCI (OPen-Label, Randomized, Controlled, Multicenter Study ExplorIng TwO TreatmeNt StratEgiEs of Rivaroxaban and a Dose-Adjusted Oral Vitamin K Antagonist Treatment Strategy in Subjects with Atrial Fibrillation who Undergo Percutaneous Coronary Intervention) trial compared low-dose rivaroxaban (15 mg once daily) plus ${\mathrm{P2Y}}_{12}$ inhibitor, or very-low-dose rivaroxaban (2.5 mg twice daily) plus DAPT, with warfarin plus DAPT to detect difference in the clinically significant bleeding between groups. Bleeding rates in the two rivaroxaban arms were significantly lower than in warfarin arm [(HR 0.59; 95% CI: 0.47–0.76) and (HR 0.63; 95% CI: 0.50–0.80), respectively]. Rates of stroke, myocardial infarction, and cardiovascular death were similar between the trial arms. Of note, the most used ${\mathrm{P2Y}}_{12}$ inhibitor was clopidogrel [50]. A post hoc analysis of the PIONEER AF-PCI trial showed that rivaroxaban in either regimen was associated with reduced recurrent hospitalization and all-cause mortality compared with traditional TAT in AF patients undergoing PCI [51]. In RE-DUAL PCI (Randomized Evaluation of Dual Antithrombotic Therapy with Dabigatran versus Triple Therapy with Warfarin in Patients with Nonvalvular Atrial Fibrillation Undergoing Percutaneous Coronary Intervention) trial, double or dual antithrombotic therapy (DAT) regimen (dabigatran plus clopidogrel or ticagrelor) showed lower major or clinically relevant non-major bleeding when compared with TAT (warfarin plus DAPT). Dabigatran 110-mg regimen was superior to TAT regimen for primary outcome (HR 0.52; 95% CI: 0.42–0.63; *p* < 0.001 for noninferiority; *p* < 0.001 for superiority), while the 150-mg regimen was non-inferior to TAT regimen (HR 0.72; 95% CI: 0.58–0.88; *p* < 0.001 for noninferiority). However, the trial was not powered to examine efficacy outcomes such as systemic thromboembolism or stent thrombosis. The comparison of dabigatran doses together with TAT showed non-inferiority in the rate of a composite of thromboembolic events, death, or unplanned revascularization (HR 1.04; 95% CI: 0.84–1.29; *p* = 0.005 for noninferiority) [52].

Apixaban was compared with VKAs in the two-by-two factorial AUGUSTUS (Aspirin Placebo in Patients with Atrial Fibrillation and Acute Coronary Syndrome or Percutaneous Coronary Intervention) trial. Patients on a ${\mathrm{P2Y}}_{12}$ inhibitor were assigned to apixaban or warfarin and aspirin or placebo. Apixaban was superior to warfarin in reducing major or clinically relevant non-major bleeding (HR 0.69; 95% CI: 0.58–0.81; *p* < 0.001 for noninferiority and superiority). Patients assigned to aspirin arm showed higher bleeding rate (HR 1.89; 95% CI: 1.59–2.24) compared with patients in placebo arm. Death or hospitalization rate was similar to that of ischemic events in aspirin and placebo groups. Apixaban showed similar ischemic outcomes to warfarin with less death or hospitalization composite events (HR 0.83; 95% CI: 0.74–0.93) [53]. The SAFE-A (SAFety and Effectiveness trial of Apixaban use in association with dual antiplatelet therapy in patients with atrial fibrillation undergoing percutaneous coronary intervention) randomized controlled trial evaluated the withdrawal of ${\mathrm{P2Y}}_{12}$ inhibitors from triple antithrombotic therapy after one or six months of therapy in AF patients undergoing PCI. The rate of primary endpoint (i.e., any bleeding) was not different between study arms. However, the enrolment of participants was slow which caused a premature trial termination [54]. In the ENTRUST-AF PCI (Evaluation of the Safety and Efficacy of an Edoxaban-Based Compared to a Vitamin K Antagonist-Based Antithrombotic Regimen in Subjects With Atrial Fibrillation Following Successful Percutaneous Coronary Intervention With Stent Placement) trial, AF patients who underwent PCI were randomly assigned to edoxaban (60 mg once daily) plus ${\mathrm{P2Y}}_{12}$ inhibitor or VKA plus DAPT. Edoxaban-regimen was non-inferior to VKA-regimen for composite of major or clinically relevant non-major bleeding (HR 0.83; 95% CI: 0.65–1.05; *p* = 0.0010 for non-inferiority) but failed to show superiority. Both regimens had similar ischemic outcomes [55]. The key studies are summarized in Table 3 (Ref. [50, 52, 53, 54, 55]).

**Table 3.** **Non-vitamin K antagonist oral anticoagulants in ischemic heart disease and atrial fibrillation**.

Study	Population	Intervention and Comparator(s)	Efficacy	Safety
NOACs vs comparator				
Acute coronary syndrome and atrial fibrillation				
Gibson *et al*. 2015 (PIONEER AF-PCI) [50]	∙ Nonvalvular AF patients who undergone PCI with stenting	∙ Group 1: Rivaroxaban 15 mg OD plus a ${\mathrm{P2Y}}_{12}$ inhibitor	MACE	Clinically significant bleeding – HR (95% CI)
RCT		∙ Group 2: Rivaroxaban 2.5 mg BID plus DAPT	Group 1 vs Group 2 vs Group 3: 6.5% vs 5.6% vs 6.0% (*p * > 0.05 for both comparisons)	∙ Group 1 vs Group 3: 16.8% vs 26.7%; HR 0.59 (0.47, 0.76)
N = 2124		∙ Group 3: VKA plus DAPT		∙ Group 2 vs Group 3: 18% vs 26.7%; HR 0.63 (0.50, 0.80)
Cannon *et al*. 2017 (RE-DUAL PCI) [52]	∙ AF patients who underwent PCI	∙ DAT: dabigatran (110 or 150 mg BID) plus ${\mathrm{P2Y}}_{12}$ inhibitor	MI, stroke, or systemic embolism, death, or unplanned revascularization – HR (95% CI)	Major or clinically relevant nonmajor bleeding – HR (95% CI)
RCT		∙ TAT: warfarin plus ${\mathrm{P2Y}}_{12}$ inhibitor and aspirin	∙ DAT (both) vs TAT: 13.7% vs 13.4%; HR 1.04 (0.84, 1.29)	∙ DAT (110 mg) vs TAT: 15.4% vs 26.9% HR 0.52 (0.42, 0.6)
N = 2725			∙ DAT (110 mg) vs TAT: 15.2% vs 13.4%; HR 1.13 (0.90, 1.43)	∙ DAT (150 mg) vs TAT: 20.2% vs 25.7%; HR 0.72 (0.58, 0.88)
			∙ DAT (150 m) vs TAT: 11.8% vs 12.8%; HR 0.89 (0.67, 1.19)	
Lopes *et al*. 2019 (AUGUSTUS) [53]	∙ AF patients with recent ACS or underwent PCI (or both) with planned ${\mathrm{P2Y}}_{12}$ inhibitor use	∙ Apixaban (5 mg or 2.5 mg BID)	Anticoagulants comparison – HR (95% CI)	Major or clinically relevant nonmajor bleeding – HR (95% CI)
2X2 factorial, RCT		∙ VKA	∙ Death or hospitalization: 23.5% vs 27.4%; HR 0.83 (0.74, 0.93)	∙ Anticoagulants comparison: 10.5% vs 14.7%; HR 0.69 (0.58, 0.81)
N = 4614		∙ Aspirin or matching placebo	∙ Death or ischemic events: 6.7% vs 7.1%; HR 0.93 (0.75, 1.16)	∙ Antiplatelets comparison: 6.1% vs 9%; HR 1.89 (1.59, 2.24)
			Antiplatelets comparison – HR (95% CI)	
			∙ Death or hospitalization: 26.2% vs 24.7%; HR 1.08 (0.96, 1.21)	
			∙ Death or ischemic events: 6.5% vs 7.3%; HR 0.89 (0.71, 1.11)	
Hoshi *et al*. 2020 (SAFE-A) [54]	∙ AF patients requiring coronary stenting	∙ Apixaban (5 mg or 2.5 mg BID) plus ${\mathrm{P2Y}}_{12}$ inhibitor and aspirin for 1 month	∙ Mortality, MI, stroke, or systemic embolization: 9.8% vs 2.8%; HR 3 (95% CI: 0.82, 10.94)	∙ Minor/major bleeding, bleeding various BARC grades or bleeding requiring blood transfusion: 11.8% vs 16.0%; HR 0.70 (95% CI: 0.33, 1.47)
RCT		∙ Apixaban (5 mg or 2.5 mg BID) plus ${\mathrm{P2Y}}_{12}$ inhibitor and aspirin for 6 months	∙ Net clinical benefit (ischemic events or bleeding: 10.8% vs 5.7%; HR 1.70 (95% CI: 0.63, 4.61)	
N = 210				
Vranckx *et al*. 2019 (ENTRUST-AF PCI) [55]	∙ AF patients underwent PCI for stable CAD or ACS	∙ Edoxaban (60 or 30 mg OD) plus ${\mathrm{P2Y}}_{12}$ inhibitor	∙ CV death, MI, stroke, systemic embolism, or definite stent thrombosis: 7.0% vs 6.0% (no difference)	∙ Major or clinically relevant nonmajor bleeding: 20.7% vs 25.6%; HR 0.83 (0.65, 1.05; *p* = 0.0010 for non-inferiority)
RCT		∙ VKA plus a ${\mathrm{P2Y}}_{12}$ inhibitor and aspirin		
N = 1506				
Chronic coronary syndrome and atrial fibrillation				
Yasuda *et al*. 2019 (AFIRE) [60]	∙ AF patients underwent PCI or CABG 1 year earlier or confirmed CAD confirmed not requiring revascularization	∙ Rivaroxaban (15 or 10 mg OD)	∙ Stroke, systemic embolism, MI, UA requiring revascularization, or death: 4.14% vs 5.75%; HR 0.72 (0.55, 0.95; *p * < 0.001 for noninferiority)	∙ Major bleeding: 1.62% vs 2.76%; HR 0.59 (95% CI: 0.39, 0.89 *p* = 0.01 for superiority)
RCT		∙ Rivaroxaban 15 mg OD plus antiplatelet (${\mathrm{P2Y}}_{12}$ inhibitor or aspirin)		
N = 2236				
Matsumura-Nakano *et al*. 2019 (OAC-ALONE) [61]	∙ AF patients beyond 1 year after stenting	∙ OAC (warfarin or dabigatran 150 or 110 mg BID, or rivaroxaban 15 or 10 mg OD, apixaban 5 or 2.5 mg BID, edoxaban 60 or 30 mg OD)	∙ Death, MI, stroke, or systemic embolism: 15.7% vs 13.6%; HR 1.16 (95% CI: 0.79, 1.72)	∙ Major bleeding: 7.8% vs 10.4%; HR 0.73 (95% CI: 0.44, 1.20)
RCT		∙ OAC and APT (aspirin or clopidogrel)	∙ Death, MI, stroke, or systemic embolism or major bleeding: 19.5% vs 19.4%; HR 0.99 (95% CI: 0.71, 1.39; *p* = 0.016 for noninferiority, *p* = 0.96 for superiority)	
N = 696				

Abbreviations: ACS, acute coronary syndrome; AF, atrial fibrillation; APT, antiplatelet agent; BARC, Bleeding Academic Research Consortium; BID, twice daily; CABG, coronary-artery bypass grafting; CAD, coronary artery disease; CI, confidence interval; CV, cardiovascular; DAPT, dual antiplatelet therapy; DAT, dual antithrombotic therapy; HR, hazard ratio; MACE, major adverse cardiovascular event; MI, myocardial infarction; NOACs, non-vitamin K oral anticoagulants; OAC, oral anticoagulants; OD, once daily; PCI, percutaneous coronary intervention; RCT, randomized controlled trials; TAT, triple antithrombotic therapy; UA, unstable angina; VKA, vitamin K antagonists.

In summary, TAT increased bleeding when compared with DAT without significant difference in mortality or stroke outcomes. Although the four trials (PIONEER AF-PCI, RE-DUAL PCI, AUGUSTUS, and ENTRUST-AF PCI) have shown the safety of DAT in the first year after PCI with regards to bleeding risk, they were not powered to assess the efficacy outcomes such as myocardial infarction, stroke, and cardiovascular death. A meta-analysis of the four randomized studies (n = 10,969) concluded that the combination of antiplatelet agents with NOACs caused lower major bleeding rates by 37% than warfarin (relative risk 0.63; 95% CI: 0.50–0.80) without increasing thrombotic or ischemic episodes [56]. Similarly, a meta-analysis (n = 10,234) showed that DAT caused lower major or clinically relevant non-major bleeding rates than TAT (risk ratio (RR) 0.66; 95% CI: 0.56–0.78) but at expense of more stent thrombosis events (RR 1.59; 95% CI: 1.01–2.50) [57]. Another meta-analysis reported similar findings where DAT was associated with less major bleeding (OR 0.598; 95% CI: 0.491–0.727) and higher stent thrombosis episodes (OR 1.672; 1.022–2.733) when compared with TAT [58]. NOAC-based DAT when compared with VKA-TAT was associated with fewer intracranial haemorrhage events (RR 0.33; 95% CI: 0.17–0.65) [57], which is consistent with lower major bleeding risk reported with NOAC-based regimens versus VKA-based regimens (OR 0.577, 0.477–0.698) [58]. Both DAT and TAT regimens showed comparable mortality and stroke rates [57, 58]. The WOEST (What is the Optimal antiplatElet & Anticoagulant Therapy in Patients With Oral Anticoagulation and Coronary StenTing) survey showed inconsistency in antithrombotic management approach by the interventional cardiologists which reflected the inconsistency between guidelines [59].

## 6. Chronic Coronary Syndrome and Atrial Fibrillation

The first trial to compare rivaroxaban alone with rivaroxaban plus single or dual antiplatelet agent(s) in stable CAD was the Japanese AFIRE (Atrial Fibrillation and Ischemic Events with Rivaroxaban in Patients with Stable Coronary Artery Disease) trial. Stable CAD was defined as undergoing PCI or coronary artery bypass grafting (CABG) more than one year earlier or having confirmed CAD not requiring revascularization. Monotherapy with rivaroxaban was non-inferior to DAT for composite of death, myocardial infarction, stroke, systemic embolism, or unstable angina requiring revascularization (HR 0.72; 95% CI: 0.55–0.95) and was superior for major bleeding (HR 0.59; 95% CI: 0.39–0.89). The trial was terminated early due to the surprisingly increased death in the combination group (Table 3) [60]. While AFIRE trial is the only one powered to detect efficacy outcomes, it is necessary to note the dissimilarities in comparison with earlier trials in ACS such as enrolment of only Japanese participants, dose of rivaroxaban adopted (i.e., 10 or 15 mg as approved in Japan), and patients were with stable CAD. Another Japanese study is the OAC-ALONE (Optimizing Antithrombotic Care in Patients With Atrial Fibrillation and Coronary Stent) trial that also examined oral anticoagulants alone in comparison with the combination therapy of an oral anticoagulant and an antiplatelet agent but only 26% of patients were on NOACs [61]. The study did not demonstrate the inferiority of oral anticoagulation monotherapy to combined therapy in patients with concurrent AF and stable CAD after one year of PCI. However, the study was underpowered due to premature enrolment termination (Table 3) [61].

Wernly *et al*. [62] have pooled the outcomes data of both AFIRE and OAC-ALONE trials (n = 1905) without finding a difference between NOACs monotherapy and combination therapy groups in term of rates of major adverse cardiovascular events, myocardial infarction, or ischemic stroke. NOACs monotherapy, however, caused less major bleeding complications (risk rate 0.66; 95% CI: 0.49–0.91) than the combination group. An observational analysis of AF patients, from Medicare American population, with documented peripheral or coronary artery disease and have newly initiated NOACs or warfarin prescription, was performed. In comparison with warfarin, patients on NOACs had lower events rate of composite of death, stroke, or myocardial infarction [(HR 0.63; 95% CI: 0.58–0.69 for apixaban), (HR 0.79; 95% CI: 0.70–0.90 for dabigatran), (HR 0.87; 95% CI: 0.81–0.92 for rivaroxaban)]. Moreover, the rate of combined systemic embolism and stroke was significantly lower with apixaban or rivaroxaban [(HR 0.48; 95% CI: 0.37–0.62) or (HR 0.72; 95% CI: 0.60–0.89), respectively]. The rate of major bleeding was lower with apixaban (HR 0.66; 95% CI: 0.58–0.75), but higher with rivaroxaban (HR 1.14; 95% CI: 1.05–1.23) in comparison with warfarin [63]. Interestingly, warfarin was associated with significant progression of coronary total and calcified plaques volumes in patients with AF as compared with rivaroxaban (20 mg daily), when evaluated by coronary computed tomography angiography in a prospective randomized study [64].

## 7. Periprocedural Management 

The adequateness of NOACs efficacy during angioplasty has been reported with conflicting findings between studies. A preclinical study demonstrated that peak dabigatran levels were insufficient to inhibit catheter-induced thrombosis unless additional heparin is administered [65]. Vranckx *et al*. [66] investigated the efficacy of dabigatran in suppressing coagulation during elective angioplasty in patients who were using NOACs for a long period. In an exploratory Phase II study (n = 50), pre-procedural 110-mg or 150-mg twice daily dabigatran in comparison with standard heparin regimen did not sufficiently suppress coagulation during PCI. The insufficient effect was evident by elevated prothrombin fragment 1+2 and thrombin-antithrombin complexes levels, in addition to more bailout anticoagulants required with dabigatran because of adverse clinical outcomes (e.g., stent thrombosis and myocardial infarction) [66]. On the other hand, data from the Dresden NOAC registry showed that either short-term interruption or continuation of NOACs during invasive procedures was safe [67]. Furthermore, Vranckx *et al*. [68] found that rivaroxaban (either 10 or 20 mg with or without heparin) was more effective in suppressing coagulation than standard heparin during angioplasty in the X-PLORER trial (Exploring the Efficacy and Safety of Rivaroxaban to Support Elective Percutaneous Coronary Intervention), an exploratory Phase II trial (n = 108). There were low levels of prothrombin fragment 1+2 and thrombin-antithrombin complexes without bailout anticoagulation, thrombotic or bleeding events with rivaroxaban [68].

## 8. In-Stent Thrombosis 

The incidence of in-stent thrombosis after angioplasty usually ranges between 0.6% and 3.3% at up to one year of follow-up, regardless of the stent type. In high-risk population, the incidence may be higher after a drug-eluting stent implantation; 2.7% within one month and ranging from 5.2% to 8.3% at 1–5 years of follow-up, respectively. Although the incidence is considered relatively low, mortality has been reported in approximately 10% to 25% of affected patients at one-year follow-up. The formed in-stent thrombi contain both platelets and fibrin suggesting that the platelet activation and thrombin generation sequence resemble thrombus formation in ACS [69]. In APPRAISE-2 trial, the incidence of stent thrombosis did not significantly differ between apixaban and placebo arms. However, the trial was terminated early because of excessive bleeding with apixaban [21]. On the other hand, rivaroxaban, in ATLAS ACS 2-TIMI 51, decreased stent thrombosis events by 31% (HR 0.69; 95% CI: 0.51–0.93) [25]. This benefit was confirmed when the outcomes of only stented patients in ATLAS ACS 2-TIMI 51 trial were analysed separately (HR 0.65; *p* = 0.017). When breaking down the results according to rivaroxaban dose, the 2.5-mg twice-daily dose reduced definite or probable stent thrombosis events (HR 0.61; *p* = 0.023), a benefit that was not observed with 5-mg twice-daily dose (*p* = 0.89). In addition, twice-daily 2.5-mg dose showed favourable mortality outcome as well (HR 0.56; 95% CI: 0.35–0.89). However, reduction in stent thrombosis by combined rivaroxaban doses was not maintained beyond the active DAPT duration, i.e., in participants on aspirin as single antiplatelet (HR 0.68; 95% CI: 0.50–0.92). Thus, rivaroxaban may only be effective with DAPT (Table 4, Ref. [21, 25, 70]) [70]. A preclinical study that examined rivaroxaban alone or combined with DAPT reported consistent results [71].

**Table 4.** **Non-vitamin K antagonist oral anticoagulants for in-stent thrombosis and after surgery**.

Study	Population	Intervention and comparator(s)	Efficacy	Safety
NOACs vs comparator				
In-stent thrombosis				
Alexander *et al*. 2011 (APPRAISE-2) [21]	∙ Recent ACS (STEMI, NSTEMI, UA) and at least two risk factors for recurrent ischemic events	∙ Apixaban 5 mg BID	∙ Stent thrombosis: 0.9% vs 1.3%, HR 0.73 (95% CI: 0.47, 1.12)	∙ Major bleeding: 1.3% vs 0.5%; HR 2.59 (95% CI: 1.50, 4.46)
RCT		∙ Placebo		∙ ICH: 0.3% vs 0.1%, HR 4.06 (95% CI: 1.15, 14.38)
N = 7392				
Mega *et al*. 2012 (ATLAS ACS 2-TIMI 51) [25]	∙ ACS (STEMI, NSTEMI, UA)	∙ Rivaroxaban 2.5 mg or 5 mg BID on top of DAPT	Stent thrombosis	Major bleeding (non-CABG)
RCT		∙ Placebo (i.e., DAPT only)	∙ 2.5 mg: 2.2% vs 2.9%; HR 0.65 (95% CI: 0.45, 0.94)	∙ 2.5 mg: 1.8% vs 0.6%; HR 3.46 (95% CI: 2.08, 5.77)
N = 15,526			∙ 5 mg: 2.3% vs 2.9%; HR 0.73 (95% CI: 0.51, 1.04)	∙ 5 mg: 2.4% vs 0.6%; HR 4.47 (95% CI: 2.71, 7.36)
			∙ Combined: 2.3% vs 2.9%; HR 0.69 (95% CI: 0.51, 0.93)	∙ Combined: 2.1% vs 0.6%; HR 3.96 (95% CI: 2.46, 6.38)
				ICH: significantly higher with rivaroxaban at all doses
Gibson *et al*. 2013 (ATLAS-ACS-51 analysis) [70]	∙ Patients with a history of stent placement	∙ Rivaroxaban 2.5 mg or 5 mg BID on top of DAPT	∙ Stent thrombosis: significantly reduced with rivaroxaban at all doses	-
N = 9631		∙ Placebo (i.e., DAPT only)	∙ Mortality (2.5 mg): HR 0.56 (95% CI: 0.35, 0.89)	
Post CABG				
Lamy *et al*. 2019 COMPASS-CABG (sub-study) [72]	∙ COMPASS trial patients 4 to 14 days after CABG surgery	∙ Rivaroxaban 2.5 mg BID plus aspirin	Rivaroxaban-plus-aspirin	-
N = 1448		∙ Rivaroxaban 5 mg BID alone	∙ Graft failure: 9.1% vs 8%; OR 1.13 (95% CI: 0.82, 1.57)	
		∙ Aspirin alone	∙ MACE: 2.4% vs 3.5%; HR 0.69 (95% CI: 0.33, 1.47)	
			Rivaroxaban vs aspirin	
			∙ Graft failure: 7.8% vs 8%; OR 0.95 (95% CI: 0.67, 1.33)	
			∙ MACE: 3.3% vs 3.5%; HR 0.99 (95% CI: 0.50, 1.99)	
MI after non-cardiac surgery				
Devereaux *et al*. 2018 (MANAGE) [73]	∙ Patients underwent non-cardiac surgery within 35 days of MINS	∙ Dabigatran 110 mg BID	∙ CV mortality, MI, stroke, peripheral arterial thrombosis, amputation or VTE: 11% vs 15%; HR 0.72 (95% CI: 0.55, 0.93)	∙ Life-threatening, major, or critical organ bleeding: 3% vs 4%; HR 0.92 (95% CI: 0.55, 1.53)
RCT		∙ Placebo		
N = 1754				

Abbreviations: ACS, acute coronary syndrome; BID, twice daily; CABG, coronary artery bypass grafting; CI, confidence interval; CV, cardiovascular; DAPT, dual antiplatelet therapy; HR, hazard ratio; ICH, intracranial haemorrhage; MACE, major adverse cardiovascular events; MI, myocardial infarction; MINS, myocardial infarction after non-cardiac surgery; NOACs, non-vitamin K antagonist oral anticoagulants; NSTEMI, non-ST segment elevation myocardial infarction; OR, odds ratio; RCT, randomized controlled trial; STEMI, ST-segment elevation myocardial elevation; UA, unstable angina; VTE, venous thromboembolism.

## 9. Post Coronary Artery Bypass Grafting 

Early graft failure after CABG surgery occurs in 30% of patients. Lamy *et al*. [72] conducted a pre-planned sub-study (n = 1448) of COMPASS trial (COMPASS-CABG) to examine rivaroxaban (either alone or combined with aspirin) in preventing early graft failure post CABG procedure. Rivaroxaban regimens did not lower the graft failure rate but only 2.5-mg twice-daily rivaroxaban dose combined with aspirin was associated with lower major adverse cardiovascular events than aspirin alone (HR 0.69; 95% CI: 0.33–1.47) (Table 4) [72].

## 10. Myocardial Injury after Noncardiac Surgery

Myocardial injury after non-cardiac surgery (MINS), defined as myocardial infarction coupled with isolated ischemic cardiac troponin rise, usually occurs within 30 days following surgery and should not comprise non-ischemic causes such as AF, sepsis, or pulmonary embolism. MINS is correlated with a four-fold increased death rate at 30 days and increased death and cardiovascular complications at two years after surgery. Devereaux *et al*. [73] in their MANAGE (Management of myocardial injury After NoncArdiac surGEry) trial (n = 1754) concluded that dabigatran (110 mg twice daily) lowered major vascular complications (11% vs 15%, HR 0.72; 95% CI: 0.55–0.93) without an increase in bleeding (3% vs 4%, HR 0.92; 95% CI: 0.55–1.53) (Table 4).

## 11. Left Ventricular Thrombus

The formation of LV thrombus following acute myocardial infarction is a common complication. The incidence rate has varied greatly in literature (2.7–43%) with higher incidence rates occurring after anterior wall myocardial infarction [5, 74]. The decreasing trends in LV thrombus incidence reflects the improvement of coronary revascularization interventions [5, 74, 75], and the contemporary antithrombotic therapies in myocardial infarction [75]. LV thrombus pathogenesis is commonly described as an interplay of Virchow’s triad factors, i.e., endocardial injury, stasis, and hypercoagulability state [74]. The formation of LV thrombus usually occurs in the first two weeks post myocardial infarction [74, 76, 77]; 24% within 24 hours, 57% within 48–72 hours, 75% and 96% at one and two week(s), respectively [78].

### 11.1 Prevention of LV Thrombus

The routine short-term use of anticoagulants to prevent the formation of LV thrombus after myocardial infarction should be individualized, considering the advantages and disadvantages of this approach as it is not supported by robust evidence. Published observational studies did not show benefit in term of major adverse cardiovascular events, rather increased major bleeding episodes. VKAs, particularly warfarin, have been the traditional agents of choice [74]. Recently, an open-label study (n = 279) by Zhang *et al*. [79], supported the 30-day use of low-dose (i.e., 2.5 mg twice daily) rivaroxaban on top of DAPT to decrease the chance of LV thrombus formation following anterior myocardial infarction compared with DAPT alone (0.7% vs 8.6%; HR 0.08; 95% CI: 0.01–0.62), without increasing bleeding risk between the study arms at the pre-specified follow-up periods (Table 5, Ref. [79]).

**Table 5.** **Non-vitamin K antagonist oral anticoagulants for prophylaxis and treatment of left ventricular thrombus**.

Study	Population	Intervention and comparator(s)	Efficacy	Safety
NOACs vs comparator				
Prophylaxis				
Zhang *et al*. 2022 [79]	∙ Patients with anterior STEMI who underwent PPCI	∙ Rivaroxaban 2.5 mg BID for 30 days and DAPT	∙ LVT formation within 30 days: 0.7% vs 8.6%; HR: 0.08 (95% CI: 0.01, 0.62)	∙ Bleeding: no difference
RCT		∙ DAPT only	∙ Net clinical adverse events were lower within 30 days with rivaroxaban and remained relatively low at 180 days	∙ ICH: 1 patient vs 0 within 30 days
N = 279				
Treatment				
Abdelnabi *et al*. 2021 (No-LVT Trial) [89]	∙ Patients with newly diagnosed LVT	∙ Rivaroxaban 20 mg OD	∙ Complete LVT resolution: at 1 month: 71.79 vs 47.5%, *p* = 0.03 (NS after adjustment); at 3 and 6 months: no difference	∙ Major bleeding: 5.1% vs 15%, *p* = 0.11
RCT		∙ Warfarin	∙ Stroke: no difference	
N = 79			∙ Systemic embolism: no difference	
			∙ Composite of both: 0 vs 15%, *p* = 0.01	
Alcalai *et al*. 2022 [90]	∙ Patients with LVT 1–14 days after acute MI	∙ Apixaban	∙ LVT resolution at 3 months: no difference	∙ Major bleeding: 0 vs 2 patients
RCT		∙ Warfarin	∙ Stroke: 0 vs 1 patient	
N = 35			∙ Death: 1 patient vs 0	
Isa *et al*. 2020 [91]	∙ Patients diagnosed with LVT	∙ Apixaban	∙ Percentage of reduction or total LVT resolution at 12 weeks: 65.1% vs 61.5%, *p* = 0.816	∙ Safety outcomes: no difference
RCT, pilot		∙ Warfarin		
N = 27				

Abbreviations: BID, twice daily; CI, confidence interval; DAPT, dual antiplatelet therapy; ICH, intracranial haemorrhage; LVT, left ventricular thrombus; NOACs, non-vitamin K oral anticoagulants; NS, not significant; OD, once daily; PPCI, primary percutaneous coronary intervention; RCT, randomized controlled trials; STEMI, ST-segment elevation myocardial infarction.

### 11.2 Treatment of LV Thrombus

The formed LV thrombus following myocardial infarction is a source of further thromboembolic events with an estimated increase in risk by 5.5 folds in comparison with no thrombus. If left untreated, the annual rate of systemic embolization and stroke is approximately 10% to 15% [74]. Moreover, the presence of LV thrombus may increase mortality risk. LV thrombus regression due to the use of anticoagulation therapy reduced mortality [80]. International guidelines consider VKAs as the first-choice treatment for LV thrombus, with a little guidance on NOACs use as alternative therapeutic option instead of warfarin in this scenario [74]. The off-label NOACs use in treating LV thrombus has been increasing substantially since 2020 [81]. Earlier reports were limited to case reports or series, their meta-summaries [82, 83], or centres experience [84]. In a meta-summary of case reports, rivaroxaban use accounted for 47.2% of NOACs use whereas 27.8% of patients used dabigatran and 25% used apixaban [82]. LV thrombus resolution occurred in 88% to 92% of patients within a median of 30–32 days [82, 83]. Overall, NOACs seemed effective and safe in treating patients with LV thrombus [82, 83, 84].

Most of the subsequent recent reports from observational studies [5, 85, 86, 87, 88], randomized controlled trials [89, 90, 91] and meta-analyses [74, 92, 93, 94, 95, 96, 97, 98, 99, 100, 101] showed consistent results. In four observational retrospective studies, the ischemic aetiology behind the thrombus formation was reported in more than 50% of the patients [85, 86, 87, 88]. VKAs and NOACs use ranged from 58% to 81% and 19% to 70%, respectively [85, 86, 87, 88], without a difference in the thromboembolic event rates except in one study that showed a significant correlation of NOACs use with systemic embolism or stroke rate (HR 2.64; 95% CI: 1.28–5.43) [88]. The LV thrombus resolution rates that were reported in two studies [85, 87] did not find difference between VKAs and NOACs groups (71.4% vs 70.6%, *p* = 0.9) [87] and (63% vs 53%, *p* = 0.85) [85]. Jones and colleagues [5] in their observational study recruited only patients with LV thrombus formation after acute myocardial infarction (n = 101). Patients on VKAs and NOACs accounted for 60% and 40% of patients, respectively. NOACs were more effective than VKAs in resolving the LV thrombus (82% vs 64.4%, *p* = 0.0018; OR 1.8; 95% CI: 1.2–2.9) with lower major bleeding rate (0% vs 6.7%, *p* = 0.030) and no difference in thromboembolic events rates (5% vs 2.4%, *p* = 0.388) [5]. Three trials that randomized patients with LV thrombus to either NOACs (apixaban or rivaroxaban) or VKAs collectively concluded that NOACs are as effective as warfarin (Table 5) [89, 90, 91]. A meta-analysis pooled data of the three randomized trials (n = 139) did not find statistical difference between NOACs and VKAs in LV thrombus resolution, mortality, and stroke but NOACs caused significantly lower major bleeding than VKA (OR 0.16; 95% credible interval: 0.02–0.82) [92]. Additional 10 meta-analyses [74, 93, 94, 95, 96, 97, 98, 99, 100, 101] showed at least comparable results between NOACs and VKAs in terms of efficacy and safety (Table 6, Ref [74, 92, 93, 94, 95, 96, 97, 98, 99, 100, 101]). One of the meta-analyses showed better resolution of LV thrombus with NOACs [97], two others found significant reduction in stroke with NOACs [93, 100], and in other two there was lower bleeding risk with NOACs [94, 99].

**Table 6.** **Meta-analyses of non-vitamin K antagonist oral anticoagulants studies in left ventricular thrombus**.

Study	Studies Population	LV thrombus resolution	Efficacy	Safety	Conclusion
NOACs vs comparator					
Chen *et al*. 2021 [93]	∙ 13 observational	∙ RR 0.88 (95% CI: 0.72, 1.09)	∙ Stroke or systemic embolism: RR 0.96 (95% CI: 0.80, 1.16)	∙ Bleedings: RR 0.94 (95% CI: 0.67, 1.31)	∙ NOACs had similar efficacy and safety profile compared with warfarin in patients with LVT
	· N = 2467		∙ Stroke: RR 0.68 (95% CI: 0.47, 1.00)	∙ Clinically relevant bleedings: RR 0.35 (95% CI: 0.13, 0.92)	∙ NOACs reduced strokes and clinically relevant bleedings
Chen *et al*. 2022 [94]	∙ 3 randomized, 12 cohort	∙ RR 1.01 (95% CI: 0.93, 1.09)	∙ Stroke and/or systematic embolic events: RR 0.87 (95% CI: 0.11, 1.55)	∙ Clinically significant bleeding: RR 0.6 (95% CI: 0.39, 0.90)	∙ NOACs were noninferior to warfarin in LVT treatment
	∙ N = 2334		∙ Mortality: RR 0.9 (95% CI: 0.58, 1.4)		∙ NOACs had lower risk of clinically significant bleeding
Cochran *et al*. 2021 [95]	∙ 6 retrospective	∙ 86 vs 76%, *p* = 0.499	∙ Strokes: 0 vs 15%, *p* = 0.189	∙ Bleeding: 14 vs 14%, *p* = 1	∙ Similar efficacy and safety of NOACs compared to VKA in treating LVT
	∙ N = 1615	∙ Unresolved thrombus: OR 0.61 (95% CI: 0.26, 1.41)	∙ ACS: 7 vs 3.4%, *p* = 0.477	∙ Bleeding events: OR 1.13 (95% CI: 0.74, 1.72)	
			∙ Embolic events: OR 1.24 (95% CI: 0.90, 1.69)		
			∙ Embolic events and death: OR 1.10 (95% CI: 0.84, 1.45)		
Dalia *et al*. 2021 [96]	∙ 8 retrospective	∙ OR 1.11 (95% CI: 0.51, 2.39)	∙ Stroke or systemic embolization: RR 1.04 (95% CI: 0.64, 1.68)	∙ Bleeding: RR 1.15 (95%: CI: 0.62, 2.13)	∙ NOACs were at least as effective as warfarin in treating of LVT without difference in stroke or bleeding events
	∙ n = 1955		∙ Mortality: RR 1.09 (95% CI: 0.70, 1.70)		
Fang *et al*. 2022 [97]	∙ 12 observational	∙ OR 1.15 (95% CI: 0.54, 2.45)	∙ Mortality: OR 0.91 (95% CI: 0.50, 1.65)	∙ Bleeding: OR 0.78 (95% CI: 0.45, 1.35)	∙ There was no difference in safety and efficacy between NOACs and VKA in patients with LVT
	∙ N = 2262		∙ SSE: OR 1.01 (95% CI: 0.66, 1.54)	Acute MI subgroup:	∙ NOACs might be superior to VKA in treating LVT in post-acute MI patients
			Acute MI subgroup:	∙ Bleeding: OR 0.38 (95% CI: 0.18, 0.81)	
			∙ SSE: OR 0.24 (95% CI: 0.07, 0.87)		
Ferreira *et al*. 2022 [98]	∙ 1 randomized, 13 retrospective	∙ OR 1.14 (95% CI: 0.77, 1.66)	∙ Efficacy outcomes: OR 0.86 (95% CI: 0.55, 1.33)	∙ Safety outcomes: OR 1.0 (95% CI: 0.78, 1.30)	∙ NOACs may have potential utility as a first-line therapy in patients with LVT
	∙ N = 2498		∙ Mortality: OR 0.92 (95% CI: 0.58, 1.45)		
Kido *et al*. 2021 [99]	∙ 8 retrospective	∙ OR 1.13 (95% CI: 0.75, 1.71)	∙ SSE: OR 0.89 (95% CI: 0.46, 1.71)	∙ Bleeding: OR 0.61 (95% CI: 0.40, 0.93)	∙ NOACs may be an alternative to VKA in treating LVT
	∙ N = 1892				
Levine *et al*. 2022 [74]	∙ 3 randomized, 18 observational	∙ OR 1.21 (95% CI: 0.89, 1.64)	∙ SSE: OR 0.94 (95% CI: 0.70, 1.25)	∙ Bleeding: OR 0.79 (95% CI: 0.56, 1.11)	∙ NOACs may be an alternative therapy in treating LVT
	∙ N = 3057		∙ Mortality: OR 0.92 (95% CI: 0.64, 1.30)		
Michael *et al*. 2021 [100]	∙ 2 randomized and 16 cohort	∙ OR 1.29 (95% CI: 0.83, 1.99)	∙ Stroke: OR 0.63 (95% CI: 0.42, 0.96)	∙ Bleeding: OR 0.72 (95% CI: 0.50, 1.02)	∙ There was reduction in stroke with NOACs use, without an increase in bleeding
	∙ N = 2666		∙ Systemic embolism: OR 0.77 (95% CI: 0.41, 1.44)		∙ NOACs may be a reasonable option in treating LVT
			∙ SSE: OR 0.83 (95% CI: 0.53, 1.33)		
			∙ Mortality: OR 1.01 (95% CI: 0.64, 1.57)		
Salah *et al*. 2021 [101]	∙ 1 randomized, 11 observational	∙ RR 0.97; 95% CI: 0.93, 1.02	∙ SSE: RR 0.95 (95% CI: 0.63, 1.45)	∙ Bleeding: RR 1.14 (95% CI: 0.81, 1.60)	∙ NOACs and warfarin had comparable efficacy and safety in treating LVT
	∙ N = 2322		∙ Mortality: RR 0.99 (95% CI: 0.67, 1.46)		
Sayed *et al*. 2021 [92]	∙ 3 randomized	∙ OR 1.17 (95% CrI: 0.37, 3.45)	∙ Mortality: OR 0.68 (95% CrI: 0.10, 4.43)	∙ Major bleeding: OR 0.16 (95% CrI: 0.02, 0.82)	∙ Results support NOACs use over warfarin in treating LVT
	∙ N = 139		∙ Stroke: OR 0.14 (95% CrI: 0.01, 1.27)		

Abbreviations: CI, confidence interval; CrI, credible interval; LVT, left ventricular thrombus; NOACs, non-vitamin K oral anticoagulants; OR, odds ratio; RR, risk ratio; SSE, stroke or systemic embolism; VKA, vitamin K antagonists.

## 12. Debatable Considerations 

### 12.1 Low-Dose NOACs

The addition of an oral anticoagulant agent to the pharmacological management of ACS has been promising particularly with the use of low-dose regimen to optimize benefit and reduce bleeding risk [2]. However, NOACs studies in patients with AF and undergoing PCI were powered for safety rather than efficacy outcomes. Thus, the protection against stroke in AF patients presenting with ACS or undergoing PCI is undetermined and may be unsuitable [102]. Rubboli *et al*. [103] examined the interpretation of lower NOACs doses in non-valvular AF by distributing a 14-statement questionnaire to physicians of different specialties. There was a wide agreement regarding the clinical implications of using lower factor Xa inhibitors doses but not dabigatran doses [103]. Cappato *et al*. [104] evaluated NOACs dose selection on all-cause mortality risk by pooling data from four major trials including ATLAS ACS-2 TIMI 51, REDEEM, COMPASS, and CAD sub-study of edoxaban landmark study in AF (n = 49,125), in which all patients had established atherosclerosis. Lower NOACs dose, but not higher NOACs dose (RR 0.95; 95% CI: 0.87–1.05), was associated with significantly lower all-cause mortality rate (RR 0.80; 95% CI: 0.73–0.89) than with control. In addition, when comparing lower versus higher NOACs dose, the benefit of lower dose was confirmed (RR 0.84; 95% CI: 0.76–0.93) [104]. Szapáry *et al*. [105] in their meta-analysis of 15 randomized studies (n = 73,536) analysed the efficacy and safety of the therapeutic options. The risk of major adverse cardiac events was significantly reduced with apixaban and dabigatran use [(RR 0.75; 95% CI: 0.58–0.98) and (RR 0.56; 95% CI: 0.39–0.80), respectively] and not with edoxaban, rivaroxaban, or VKAs use. Their use was associated with significant increase in risk of bleeding (RR 5.47, 3.66, or 1.66, respectively). When reducing NOACs dose, there was a non-significant tendency of reduced bleeding but increased risk of major adverse cardiac events [105].

### 12.2 NOACs in Combination with Antiplatelet Therapy 

The evidence on efficacy of NOACs when combined with antiplatelet therapy is still conflicting. Szapáry *et al*. [105] in their meta-analysis analysed the use of NOACs with aspirin which did not reduce risk of major adverse cardiac events but was associated with a trend towards non-significant increase in risk of bleeding (66%). As low-dose rivaroxaban combined with aspirin and clopidogrel aimed to lower cardiovascular adverse events in ACS patients, intensification of antiplatelet regimen by using ticagrelor or prasugrel instead of clopidogrel may also enhance efficacy but warrant investigation [2]. The components and optimal duration of thrombotic regimen (i.e., DAT or TAT) in ACS patients with or without AF is still debatable [106]. A post hoc analysis of the AUGUSTUS trial reported that the use of aspirin for up to 30 days after ACS resulted in more bleeding but fewer ischemic events (i.e., equal trade-off) than placebo. Whereas its use after 30 days and up to six months caused more bleeding but similar ischemic event rates [107]. In AF patients of 65 years of age or older who underwent PCI (n = 4959), Hess *et al*. [108] found that 27.6% of patients were discharge on TAT. In comparison with DAPT, patients who received TAT experienced significantly more bleeding that required hospitalization (adjusted HR: 1.61; 95% CI: 1.31–1.97) or intracranial haemorrhage (adjusted HR: 2.04; 95% CI: 1.25–3.34) without a difference in risk of major adverse cardiac events (adjusted HR 0.99; 95% CI: 0.86–1.16) [108]. Overall, it is acceptable to consider one-week duration of TAT in AF patients with low ischemic risk who underwent uncomplicated PCI and longer period (e.g., four to six weeks) for patients with higher thrombotic risk. The subsequent DAT may be continued for six to 12 months according to patients risk factors [106].

### 12.3 Risk of Myocardial Infarction

NOACs may have a more balanced benefit-risk profile in comparison with warfarin. However, the RE-LY (Randomized Evaluation of Long-term Anticoagulant Therapy) trial in atrial fibrillation has reported higher rate of myocardial infarction with dabigatran than warfarin [(relative risk 1.35; 95% CI: 0.98–1.87 for 110-mg), (relative risk 1.38; 95% CI: 1.00–1.91 for 150-mg regimen)] [109]. In RE-DUAL PCI trial, there was a non-significant higher myocardial infarction rate in dabigatran group. However, the study was not powered to detect a difference in ischemic episodes between the study arms [52]. In contrast, there was numerically lower myocardial infarction events rate with factor Xa inhibitors use [110]. A meta-analysis of nine trials (n = 53,827) in any indication for NOACs, concluded that rivaroxaban was correlated with significantly reduced myocardial infarction risk (OR 0.82; 95% CI: 0.72–0.94) in comparison with any control (i.e., warfarin, enoxaparin, or placebo) which was confirmed by trial sequential analysis [111]. Real-world evidence has not confirmed the reported myocardial infarction risk with dabigatran use [112]. As an example, Lee *et al*. [113] used the Danish registers to investigate the risk of myocardial infarction in association with NOACs and VKAs use in patients with AF (n = 31,739). Standardized one-year risk of myocardial infarction was 1.6% (95% CI: 1.3–1.8), 1.2% (95% CI: 0.9–1.4), 1.2% (95% CI: 1.0–1.5), and 1.1% (95% CI: 0.8–1.3) for VKAs, apixaban, dabigatran, and rivaroxaban, respectively. When performing various comparisons, there were not differences in myocardial infarction risk in the direct comparisons between individual NOACs, and in comparison with VKAs, all NOACs were associated with significantly lower risk [113]. On the other side, evidence from meta-analyses reported an increased myocardial infarction risk in association with dabigatran use specifically [114, 115, 116]. Kupó *et al*. [117] pooled the data of 28 randomized trials (n = 196,761) in a network meta-analysis and demonstrated that in comparison to dabigatran, rivaroxaban (relative risk 0.70; 95% credible interval (CrI): 0.53–0.89), apixaban (0.76; 95% CrI: 0.58–0.99), or VKAs (0.81; 95% CrI: 0.65–0.98) use was correlated with reductions in the relative risk of myocardial infarction. In addition, rivaroxaban was also associated with myocardial infarction risk reduction in comparison to placebo (relative risk 0.79; 95% CrI: 0.65–0.94) and its computed probability was 61.8% as being the first or best treatment option [117]. Grajek *et al*. [112] conducted a meta-analysis of eight randomized trials (n = 81,943), two landmark Phase III trials for each of the four NOACs; one pivotal trial in AF patients and another in AF patients undergoing PCI. The rate of myocardial infarction was 2.1% of all patients. In comparison with warfarin, dabigatran was associated with a significant increase in the risk of myocardial infarction by 38% regardless of the dose, whereas factor Xa inhibitors (apixaban, edoxaban, rivaroxaban) were associated with a non-significant trend towards reducing the risk by 4–5% with a significant difference between dabigatran and factors Xa inhibitors. In addition, the authors estimated the ranking of tested agents’ effectiveness in lowering myocardial infarction risk, i.e., protection from myocardial infarction. The weakest effectiveness was for dabigatran (8% for 110-mg and 14% for 150-mg regimen) and the highest was for rivaroxaban 15 mg (90%) and apixaban 5 mg (80%), which might not support the class effect concept in the NOACs group [112]. Several mechanisms have been postulated for the increased risk of myocardial infarction in association with dabigatran which may have pro-thrombotic effects [110]. Direct thrombin inhibition by dabigatran is weaker than that of warfarin and is dependent on dabigatran’s serum level. A paradoxical generation of thrombin can occur when its level decreases. The hypercoagulability paradox may occur due to the suppression of thrombin-thrombomodulin complex and inhibition of protein C activation and hence potentiating negative feedback cycle. In the presence of increased tissue factor levels resulting from plaque rupture, thrombin-drug complex may cleave [112]. *In-vitro*, dabigatran potentiated platelet adhesion and enhanced thrombosis on human plaque material which depends on the platelet altered thrombin-glycoprotein Ibα interaction that augments von Willebrand factor binding. In addition, dabigatran may also potentiate thrombin-induced platelet aggregation. Analysis of platelets protease activated receptors (PAR) demonstrated that dabigatran can acutely inhibit thrombin-induced PAR-1 activation, cleavage, and internalization in a dose-dependent fashion [110]. Moreover, prolonged thrombin inactivation by dabigatran may enhance PAR-1-surface expression [110, 112]. Inflammatory makers may also increase during treatment with direct thrombin inhibitors [112]. Conversely, *in-vitro* rivaroxaban decreased platelet aggregation triggered by tissue-factor or thrombin receptor activating peptide [110].

## 13. Areas of Uncertainty and Future Direction

NOACs showed benefit in secondary prevention of major adverse cardiovascular outcomes after ACS given the potential role of thrombin and other relevant factors in the coagulation process. However, their benefit was counteracted by the major bleeding complications [8]. It remains uncertain whether triple therapy with low-dose rivaroxaban can be extended beyond the first year or whether low-dose rivaroxaban may be combined with DAPT using aspirin and ticagrelor/prasugrel instead of clopidogrel [6]. In the presence of comorbid AF, it is still uncertain which is the optimal antithrombotic therapy beyond 12 months following the ACS events. Currently, experts’ consensus is to continue with NOACs monotherapy after dropping the antiplatelet therapy [8]. The AQUATIC (Assessment of Quitting Versus Using Aspirin Therapy In Patients Treated With Oral Anticoagulation for Atrial Fibrillation With Stabilized Coronary Artery Disease; NCT04217447) trial may address the limitations of the published AFIRE study in patients with AF and stable CAD. In addition, the optimal duration of TAT before switching to DAT and the combination of NOACs with ${\mathrm{P2Y}}_{12}$ inhibitors remain to be confirmed [6]. The ongoing RT-AF randomized study is investigating the combination of rivaroxaban and ticagrelor in AF patients presenting with ACS and undergoing PCI (NCT02334254) [118]. The results of a feasibility study on the efficacy and safety of rivaroxaban in acute phase of ACS in comparison with enoxaparin 1 mg/kg twice daily has just been published and showed non-inferiority of rivaroxaban 5 mg twice daily to the standard subcutaneous enoxaparin. The study provided important information to design future trials with adequate sample sizes [119]. The current evidence for the use of NOACs in managing LV thrombus is limited to small-scale studies. Larger randomized trials are vital to support the effectiveness and safety of NOACs in preventing and treating LV thrombus. Three studies are testing rivaroxaban in treating LV thrombus (NCT03764241, NCT04970381, NCT04970576) and two more in preventing thrombus formation after acute myocardial infarction (NCT03786757, NCT05077683). Finally, more basic studies are also needed to confirm or refute the hypothesis of increased myocardial infarction risk in association with direct thrombin inhibition and whether the risk is reduced with factor Xa inhibition [110].

## 14. Conclusions 

Despite optimal antiplatelet therapy in ACS, cardiovascular events may recur, in part due to thrombin generation. Adjunctive NOAC therapy has the potential to prevent the formation of thrombus, in the presence or absence of AF, but at the expense of increased episodes of bleeding. NOACs have also shown a promising efficacy in the management of LV thrombus and a potential benefit in preventing stent thrombosis after PCI. Taken as a whole, NOACs are increasingly used for off-licence indications, and continue to evolve as essential therapies in preventing and treating thrombotic events. The unmet need for more active and possibly more targeted anticoagulation strategy is still a problem in the field of the treatment of ACS.

## Abbreviations

ACS, Acute coronary syndrome; AF, Atrial fibrillation; AFIRE, Atrial Fibrillation and Ischemic Events with Rivaroxaban in Patients with Stable Coronary Artery Disease; APPRAISE, Apixaban for Prevention of Acute Ischemic and Safety Events; ATLAS ACS-TIMI, Anti-Xa Therapy to Lower Cardiovascular Events in Addition to Standard Therapy in Subjects with Acute Coronary Syndrome-Thrombolysis In Myocardial Infarction; AUGUSTUS, Aspirin Placebo in Patients with Atrial Fibrillation and Acute Coronary Syndrome or Percutaneous Coronary Intervention; AXIOM ACS, Safety and efficacy of TAK-442 in subjects with acute coronary syndromes; CABG, coronary artery bypass grafting; CAD, coronary artery disease; COMMANDER-HF, A Study to Assess the Effectiveness and Safety of Rivaroxaban in Reducing the Risk of Death, Myocardial Infarction, or Stroke in Participants With Heart Failure and Coronary Artery Disease Following an Episode of Decompensated Heart Failure; COMPASS, Cardiovascular OutcoMes for People Using Anticoagulation StrategieS; CrI, credible interval; DAPT, dual antiplatelet therapy; DAT, double or dual antithrombotic therapy; ENTRUST-AF PCI, Evaluation of the Safety and Efficacy of an Edoxaban-Based Compared to a Vitamin K Antagonist-Based Antithrombotic Regimen in Subjects With Atrial Fibrillation Following Successful Percutaneous Coronary Intervention With Stent Placement; ESTEEM, Efficacy and safety of the oral direct thrombin inhibitor ximelagatran in patients with recent myocardial damage; GEMINI-ACS-1, Randomized, Double-Blind, Double-Dummy, Active-Controlled, Parallel-group, Multicenter Study to Compare the Safety of Rivaroxaban Versus Acetylsalicylic Acid in Addition to Either Clopidogrel or Ticagrelor Therapy in Subjects With Acute Coronary Syndrome; HR, hazard ratio; LA, left ventricular; MANAGE, Management of myocardial injury After NoncArdiac surgery; MINS, Myocardial injury after non-cardiac surgery; NOACs, non-vitamin K antagonist oral anticoagulants; OAC-ALONE, Optimizing Antithrombotic Care in Patients With Atrial Fibrillation and Coronary Stent; PCI, percutaneous coronary intervention; PIONEER AF-PCI, OPen-Label, Randomized, Controlled, Multicenter Study ExplorIng TwO TreatmeNt StratEgiEs of Rivaroxaban and a Dose-Adjusted Oral Vitamin K Antagonist Treatment Strategy in Subjects with Atrial Fibrillation who Undergo Percutaneous Coronary Intervention; OR, odds ratio; PAR, protease activate receptors; REDEEM, Randomized Dabigatran Etexilate Dose Finding Study in Patients with Acute Coronary Syndromes Post Index Event with Additional Risk factors for Cardiovascular Complications Also Receiving Aspirin and Clopidogrel; RE-DUAL PCI, Randomized Evaluation of Dual Antithrombotic Therapy with Dabigatran versus Triple Therapy with Warfarin in Patients with Nonvalvular Atrial Fibrillation Undergoing Percutaneous Coronary Intervention; RE-LY, Randomized Evaluation of Long-term Anticoagulant Therapy; RR, risk ratio; RUBY-1, randomized, double blind, placebo-controlled trial of safety and tolerability novel oral factor Xa inhibitor darexaban (YM150) following acute coronary syndrome; SAFE-A, SAFety and Effectiveness trial of Apixaban use in association with dual antiplatelet therapy in patients with atrial fibrillation undergoing percutaneous coronary intervention; SEPIA-ACS1 TIMI 42, Study Program to Evaluate the Prevention of Ischemia with direct Anti-Xa inhibition in Acute Coronary Syndromes 1—Thrombolysis in Myocardial Infarction 42; TAO, Treatment of Acute Coronary Syndromes with Otamixaban; TAT, triple antithrombotic therapy; VKAs, vitamin K antagonists; WOEST, What is the Optimal antiplatElet & Anticoagulant Therapy in Patients With Oral Anticoagulation and Coronary StenTing; X-PLORER trial, Exploring the Efficacy and Safety of Rivaroxaban to Support Elective Percutaneous Coronary Intervention.
